# Is Transition to Retirement Associated With Volunteering? Longitudinal Evidence from Europe

**DOI:** 10.1177/01640275241251786

**Published:** 2024-05-10

**Authors:** Hans Hämäläinen, Antti O. Tanskanen, Bruno Arpino, Aïda Solé-Auró, Mirkka Danielsbacka

**Affiliations:** 1 8058 University of Turku, Turku, Finland; 2 Population Research Institute, Väestöliitto, Helsinki, Finland; 3 9308 University of Padua, Padua, Italy; 4 16770 Pompeu Fabra University, Barcelona, Spain

**Keywords:** active aging, Europe, retirement, volunteering

## Abstract

Studies have shown that retired older adults are more likely to volunteer than their working counterparts. However, whether the transition to retirement is associated with increased volunteering frequency and whether this varies according to material and time resources of participants is unclear. We used four waves of data from the longitudinal Survey of Health, Ageing and Retirement in Europe, collected between 2011–2018 across 19 countries (*n* = 12,400 person-observations from 6200 individuals over 50). Within-person (or panel fixed-effect) regression analyses revealed that transition to retirement was associated with an increased volunteering frequency over time. This association was stronger among individuals with better health, higher education, improved financial situation and in countries with higher gross domestic product per capita. Overall, transition to retirement tends to open new ways of organizing everyday life and is associated with increased frequency of volunteering.

Active aging has become a policy priority in contemporary Western countries owing to their rapidly aging populations (Walker & Maltby, 2012). Thus, the question of how older adults organize their everyday lives after retirement has become increasingly salient. Volunteering is a key example of an active aging activity that can improve both societal welfare (Musick & Wilson, 2007) and the wellbeing of older individuals engaging in such activities (Arpino & Solé-Auró, 2019; Burr et al., 2021). Volunteering can be defined as an unpaid activity that benefits other parties with whom the individual does not have a personal relationship (Hämäläinen et al., 2023). Hence, informal help and care given to family members and friends are not considered volunteering, nor are monetary donations to charitable organizations (Wilson & Musick, 1997). As one of the most important life course events experienced in later life, the transition to retirement induces new forms of organizing life as older adults’ free time and consequently, their opportunities to engage in volunteering tend to increase (Van den Bogaard et al., 2014).

Studies have reported that retired individuals are more likely to participate in volunteering than those who are still working (e.g., Hank & Erlinghagen, 2010a; Mutchler et al., 2003), although by contrast, a few studies have not detected such association (Henning et al., 2023; Tang, 2016). Prior studies on the association between retirement and volunteering have almost exclusively compared working older adults with their retired counterparts, and there is a lack of research that has examined how the transition from paid work to retirement is associated with the frequency of volunteering within individuals’ life courses. In a recent study, Eibich et al. (2022) used an instrumental variable approach and detected support for the prediction that retirement is causally associated with increased frequency of volunteering in Europe and US. However, they did not consider whether the effect of retirement on the frequency of volunteering vary according to resources of individuals.

To fill the gaps of prior studies, we used longitudinal data from 19 European countries and executed within-person regression models to explore whether the transition to retirement is associated with an individual’s frequency of volunteering over time. Moreover, we investigated whether the effect of retirement on volunteering varies according to the resources of both individual- and at the country-level.

## Theoretical Background and Hypotheses

Resource and continuity theories have been popular in prior studies considering the association between retirement and volunteering. Continuity theory argues that individuals tend to seek stability during periods of change (Atchley, 1971, 1989), meaning that older adults will seek continuity and cohesion when transitioning from paid work to retirement (Van den Bogaard et al., 2014). The idea is not that individuals try to maintain everything as it was when they were working—which is impossible as change is unavoidable—but rather that they seek to generate consistency in their activities after they retire (Atchley, 1989). Besides being a source of income, paid work may offer an individual many important benefits. Employment provides people a concrete place to go, meaningful things to do, social ties, social status, and may even form a basis for an individual’s identity. Consequently, retirement may mean a loss of the meaningful and important advantages related to work (Van den Bogaard et al., 2014). According to continuity theory, individuals may try to compensate these losses by increasing their engagement in other activities, such as volunteering which is one potential substitute for employment after retirement (Chambre, 1984; Eibich et al., 2022), especially if the individual has previous volunteering experience (Erlinghagen, 2010).

The resource theory of volunteering, in turn, posits that when individuals have more resources, they are also more likely to volunteer (Cheng et al., 2022; Principi et al., 2016; Wilson & Musick, 1997). Available time can be considered as a crucial resource enabling volunteering. Although retirement can be a gradual process in which working hours are already reduced before full retirement (e.g., Grünwald et al., 2021), retirement typically decreases substantially the amount of time spent on paid-work and therefore increases spare time. Based on continuity and resource theories we hypothesize that:

Hypothesis 1 Transition to retirement is associated with increased frequency of volunteering.

The amount of spare time is not the only factor enabling individuals to volunteer after retirement as help-giving activities are always related to the adequacy of other resources and opportunities (e.g., Hämäläinen et al., 2023; Wilson & Musick, 1998). Previous studies have highlighted the importance of human capital (e.g., health, education and wealth) in facilitating participation in volunteering (e.g., Baik et al., 2023; Wilson & Musick, 1997). Physical health of older individuals can vary substantially, which may affect their abilities to participate in different activities (Principi et al., 2016). Indeed, prior studies have detected that better health is associated with more frequent volunteering (e.g., Hank, 2011; Papa et al., 2019). It has been also evidenced that individuals with higher education volunteer more than their lower-educated counterparts (Ajrouch et al., 2016; Hank & Erlinghagen, 2010b; Hank & Stuck, 2008; Principi et al., 2016). Advanced education may reflect better individual skills for volunteering and, on the other hand, higher education can act as a signal for organizations seeking new volunteers, leading them to recruit those with higher educational backgrounds (Brady et al., 1999). Moreover, education tends to expand one’s personal social networks, which may result in improved opportunities for volunteering (Egerton & Mullan, 2008). In addition, improved financial resources predict volunteering and individuals with higher income are more likely to volunteer than those with lower income (Choi, 2003; Principi et al., 2016). Even though, volunteering does not include giving money, more secured financial situation may nevertheless reflect individuals’ possibilities to devote more time to non-paid activities. Here, we expect that the better individuals’ resources, the stronger is the positive association between retirement and volunteering:

Hypothesis 2a Transition to retirement is associated with increased frequency of volunteering more strongly among individuals with better self-rated health.

Hypothesis 2b Transition to retirement is associated with increased frequency of volunteering more strongly among higher educated individuals.

Hypothesis 2c Transition to retirement is associated with increased frequency of volunteering more strongly among individuals with better financial condition.

Volunteering is also related to the societal context. The share of older Europeans participating in volunteer activities varies from 2% in Poland to 38% in the Netherlands, and there is a general trend indicating that people from northern and central European countries volunteer more than those from southern and eastern European countries (Morawski et al., 2020). The variation in volunteering rates among older adults aligns with the strength of a welfare state (Erlinghagen & Hank, 2006; Hank & Erlinghagen, 2010b) as well as with the countries’ wealth as measured by Gross Domestic Product (GDP) per capita (Morawski et al., 2020), indicating that better country-level resources may promote volunteering. Therefore we predict that:

Hypothesis 3 Transition to retirement is associated with increased in frequency of volunteering more strongly in countries with higher GDP per capita.

Besides volunteering, individuals typically engage in other types of activities as well, especially those highly valued by people, such as the provision of care for family members. Multiple concurrent engagements can be demanding due to resource-related constraints (Hämäläinen et al., 2023; Strauss, 2021; Tanskanen et al., 2022*), *and thus individuals may need to prioritize one activity over others*.* Grandparenthood is often a cherished stage of life, and grandparents invest a great deal of resources in their grandchildren (e.g., Di Gessa et al., 2020; Tanskanen & Danielsbacka, 2019). Grandparenthood can offer a desirable substitute for employment after retirement, meaning that transition to retirement may be more associated with increased grandparenting activities rather than involvement in volunteering. Especially in countries with limited public daycare services, families with small children may have more severe need for grandparental help and thus retirees with grandchildren may channel their time to grandparenting in the expense of other activities (Grünwald et al., 2021; Tanskanen et al., 2021). Moreover, if individuals use substiantial amount of time to caregiving towards other family members, they may have less time to volunteer. For instance, spousal care and care for older parents can be really time consuming and decrease the possibilities to invest more resources on volunteering after retirement (Baik et al., 2023; Choi et al., 2007). Thus, we assume that:

Hypothesis 4a Transition to retirement is associated with increased frequency of volunteering more strongly among individuals without grandchildren than among grandparents.

Hypothesis 4b Transition to retirement is associated with increased frequency of volunteering more strongly among individuals without within-household care duties than those with such duties.

## Methods

### Sample

We used longitudinal data drawn from the Survey of Health, Ageing and Retirement in Europe (SHARE) to study how the transition to retirement is associated with the frequency of volunteering among older adults. Using computer-assisted personal interviewing method, SHARE collects data on people aged 50 and above, who speak the official language of their country of residence, and who are not living abroad or in an institution during the fieldwork period (Börsch-Supan et al., 2013). Each participating country has designed the most appropriate probabilistic sampling method for the target population (for sampling methods, see Bethmann et al., 2019). Additionally, after the initial wave of data collection, refreshment samples were drawn in some waves to compensate for panel attrition and to mantain the samples representative of the 50+ populations (Börsch-Supan et al., 2013; for the development of panel samples, see Bergmann et al., 2022). In the present study, the sample included respondents from the fourth, fifth, sixth, and seventh waves of SHARE, which were conducted every second year between 2011 and 2018 in 19 European countries: Austria, Belgium, Croatia, the Czech Republic, Denmark, Estonia, France, Germany, Greece, Hungary, Italy, Luxembourg, the Netherlands, Poland, Portugal, Slovenia, Spain, Sweden, and Switzerland. The first and second wave data were not included in the study sample because questions about volunteering were asked in a different manner than in the later waves. Moreover, the third SHARE wave (called SHARELIFE) was excluded because it involved collecting retrospective life history information and employed a different questionnaire than the regular SHARE. The seventh wave of SHARE data collection consisted of the SHARELIFE and a condensed regular questionnaire for those who had not participated in the third wave (that was the original SHARELIFE wave), whereas the normal regular questionnaire was directed only to those who had participated in the SHARELIFE earlier. However, our variables of interest were included in both of those questionnaires (with the exception of variables measuring within-household care), and therefore we were also able to utilize the seventh wave. Finally, the eighth wave data was not used because the data collection was interrupted in its early stage by COVID-19 pandemic.

Initially, the data included 102,321 persons; however, while constructing the study sample, we made several selections. First, respondents who participated in only one study wave were excluded (*n* = 74,020). Second, respondents who were not either retired or working (e.g., unemployed, permanently sick or disabled, and homemakers) were excluded as we are interested in studying the transition from paid employment to retirement (*n* = 66,676). Finally, only respondents who were working at time 1 (before retirement) and retired at time 2 (i.e., they experienced the transition from paid work to retirement but did not move from retirement back to paid work) were included in the models. These criteria left us with a study sample of 12,400 person-observations from 6200 individuals who were 50–92 years at the time of the interviews. The distribution of the observations across the study waves is shown in Table 1.

**Table 1.** Distribution of Observations Across the Study Sample (%).

		Time 2			
		Wave 5	Wave 6	Wave 7
Time 1	Wave 4	23.8	7.4	10.4
	Wave 5		24.5	7.0
	Wave 6			27.0

*Notes*. *n* = 12,400 person-observations from 6200 unique individuals.

### Measures

*Frequency of volunteering* was used as the dependent variable. In SHARE, all the respondents were first asked whether they had undertaken voluntary or charity work in the past 12 months. Those who responded “yes” were then asked to report how often they had participated in such activity, choosing from the following responses: 1 = almost daily, 2 = almost every week, 3 = almost every month, and 4 = less often. Our dependent variable was rated on a range of 0–4: 0 = no volunteering, 1 = less than monthly, 2 = almost every month, 3 = almost every week, and 4 = almost daily.

The main independent variable was *retirement status*. In the questionnaire respondents were inquired which of the given situation best described their current employment status: 1) Retired; 2) Employed or self-employed; 3) Unemployed; 4) Permanently sick or disabled; 5) Homemaker; 6) Other. For the analyses we selected only those respondents who self-defined as employed or self-employed (0 = working) at time 1 and retired (1 = retired) at time 2.

In the questionnaire, respondents were inquired the year and month of their retirement. Unfortunately, most respondents only provided the year of retirement, and therefore we were not able to construct a variable allowing us to control for the timely order of events (i.e., retirement before volunteering). However, we did run the main analyses by restricting the sample to those respondents who had not retired in the same year as participating in the interview (leaving us with 5724 unique individuals), and because most of the interviews were conducted in the first half of the year, we further excluded those who had retired in the same or previous year of the interview (leaving us with 4141 unique individuals). When conducting sensitivity analyses using the above-mentioned restricted samples, the results remained practically the same in both cases.

As the association between retirement and volunteering frequency may vary depending on the individual- and country-level resources, we investigated the differences according to self-rated health, years of education, financial situation and country groups. In the questionnaire, respondents were asked to rate their health using a five-point scale, which we subsequently recoded into three categories: 0 = Fair at best, 1 = Good, 2 = Very good. Respondents’ years of education were classified into three categories: 1 = low (lowest 25%), 2 = medium (middle 50%), and 3 = high (highest 25%). We used information on the years of education instead of the highest degree obtained, because it offers a more comparable measurement of educational duration between individuals coming from different countries and educational systems. Regarding financial situation, respondents were asked to describe how their household manages financially using a four-point scale, and consequently the variable was recoded into three categories: 0 = With difficulty, 1 = Fairly easily, 2 = Easily. Country groups were used instead of specific countries to avoid a loss of statistical power. Countries were grouped based on their gross domestic product (GDP) per capita averaged over the study waves, adjusted to constant 2010 prices (statistics retrieved from Eurostat, 2023). Using the 33^rd^ (around 4300€) and 66^th^ (around 10 500€) percentiles as thresholds, the countries were divided into three groups: 1 = Low (i.e., lowest tertile: Croatia, the Czech Republic, Estonia, Hungary, Poland, Portugal), 2 = Medium (i.e., middle tertile: Belgium, Germany, Greece, Italy, Slovenia, Spain), and 3 = High (i.e., highest tertile: Austria, Denmark, France, Luxembourg, the Netherlands, Sweden, Switzerland) GDP per capita. For sensitivity purposes, we ran the analyses using different thresholds of grouping, which did not change the main results. In the models with interactions, education and country group were treated as time-invariant variables, that is, they were measured at time 1 (before retirement).

Moreover, as the association between retirement and volunteering frequency may vary according to caregiving towards family members, we explored the differences based on grandparenthood status (0 = No grandchildren, 1 = Has grandchildren) and within-household care responsibilities (0 = No, 1 = Yes). The last-mentioned was measured by inquiring whether respondents have provided regular care to someone living in the same household with them (e.g., parent, spouse, adult child etc.). In the seventh wave of SHARE this question was only incorporated in the regular wave, which resulted a drop in the total number of observations in our study sample, and for that reason analyses were conducted separately when considering within-household care. In addition to above mentioned time-variant variables, we controlled for change in partnership status, because previous results have indicated that it may affect the frequency of volunteering (Butrica et al., 2009; Principi et al., 2016; Rotolo & Wilson, 2006). Furthermore, as we ran within-person regression models, all time-invariant factors were considered in the design itself, as discussed in the Analysis section below. Descriptive statistics are shown in Table 2.

**Table 2.** Descriptive Statistics.

	%	Mean	Within person SD	Min–Max
Gender				
Men	52.3			
Women	47.7			
Age at interview		62.15	1.52	50–92
Self-rated health				
Fair at best	27.8			
Good	39.9			
Very good	32.3			
Partnership status				
Living without partner	25.1			
Living with partner	74.9			
Education				
Low	18.2			
Medium	46.4			
High	35.4			
Financial condition				
*Household manages financially:*				
With difficulty	28.3			
Fairly easily	30.0			
Easily	41.7			
Grandparenthood status				
No grandchildren	35.3			
Has grandchildren	64.7			
Provided care within household^a^				
No	94.8			
Yes	5.3			
Country group (GDP per capita)				
Low	32.3			
Medium	30.6			
High	37.1			
**Volunteering**		0.44	0.52	0–4

*Notes*. n = 12,400 person-observations from 6200 unique individuals.

^a^Wave 7: this question was included only in regular questionnaire; *n* = 8060 person-observations from 4030 unique individuals.

### Analysis

We executed within-person (or panel fixed effect) regression models to investigate whether the transition to retirement is associated with the frequency of volunteering. Within-person models consider person-specific changes and show a variation in an individual’s behavior over time; in this case, they display whether the transition to retirement is associated with increases or decreases in the frequency of volunteering. In within-person models, the repeated measures (i.e., person-observations) were nested within responding individuals (Jokela et al., 2018). The participants served as their own controls, and these models eliminated all time-invariant factors (Allison, 2009; Brüderl & Ludwig, 2015), meaning that the factors whose values did not change between the study waves were controlled for regardless of whether they were available in the SHARE data (e.g., stable personality traits, as well as many genetic factors and other selection effects). Total (or random effect) regression models were not used as they include both within-person and between-person variation and, thus, they may not appropriately capture the unobserved (time-invariant) heterogeneity. Moreover, as we were not interested in examining the differences between older adults who had retired and those who were still working, we excluded between-person variations and concentrated on within-person variations (Curran & Bauer, 2011; Morgan, 2013).

All statistical analyses were performed using Stata 18 software. In the tables, the magnitudes of the coefficients are presented as regression coefficients from the linear regression models. In the figures, we illustrate the results by calculating the predictive margins and 95% confidence intervals from the regression models (see Williams, 2012, for the margins command in Stata).

To gain more robust findings, we ran multiple sensitivity analyses. First, as retirement may have an influence on the participants’ self-rated health, we ran a sensitivity analysis in which the health was not controlled for. Second, as retirement can be a gradual process rather than an abrupt event of transitioning from full-time working to full retirement, we considered only those respondents who did at least one hour paid work per week, and explored whether the amount of working hours affected the association between retirement and volunteering. In addition, we examined whether the association between retirement and volunteering was different according to whether individuals retired from part- or full-time work to part-time or full retirement. Third, as we focused on determining whether the transition to retirement changes the frequency of volunteering, for sensitivity purposes, we considered only volunteering participants (i.e., whether voluntary workers intensified their frequency of volunteering as result of retirement). Fourth, as there could be a qualitative difference between those who *intensify* and those who *start* volunteering because of retirement, we ran sensitivity analysis to investigate whether older adults also start volunteering as a result of retirement (i.e., whether they change from the “no volunteering” group to the “volunteering” group. Finally, although the frequency of volunteering variable was not normally distributed, we did not use (multinomial) logit models because of their limitations (for discussion, see Mood, 2010). Instead, we executed sensitivity analyses using logistic regression with different cut-off points. For the logistic regression models, we constructed three dichotomous volunteering variables: 0 = no volunteering, 1 = at least some volunteering (including all other classes) (this variable was used in the analysis to test whether individuals start volunteering as a result of retirement); 0 = less often than almost monthly, 1 = at least almost monthly; and 0 = less often than almost every week, 1 = almost daily or every week.

## Results

According to the transition probabilities of the frequency of volunteering, a significant number of older adults remained in the same category, and when changes in the frequency of volunteering occurred, there was more often a transition between categories close to each other than those further apart (Appendix Table 1). Stability and changes in volunteering frequencies were indicated by intraclass correlations that report the correlation of person-observations for an individual over time. The intraclass correlation for the frequency of volunteering was 0.59, which indicated a moderate stability between study waves.

**Figure 1.**
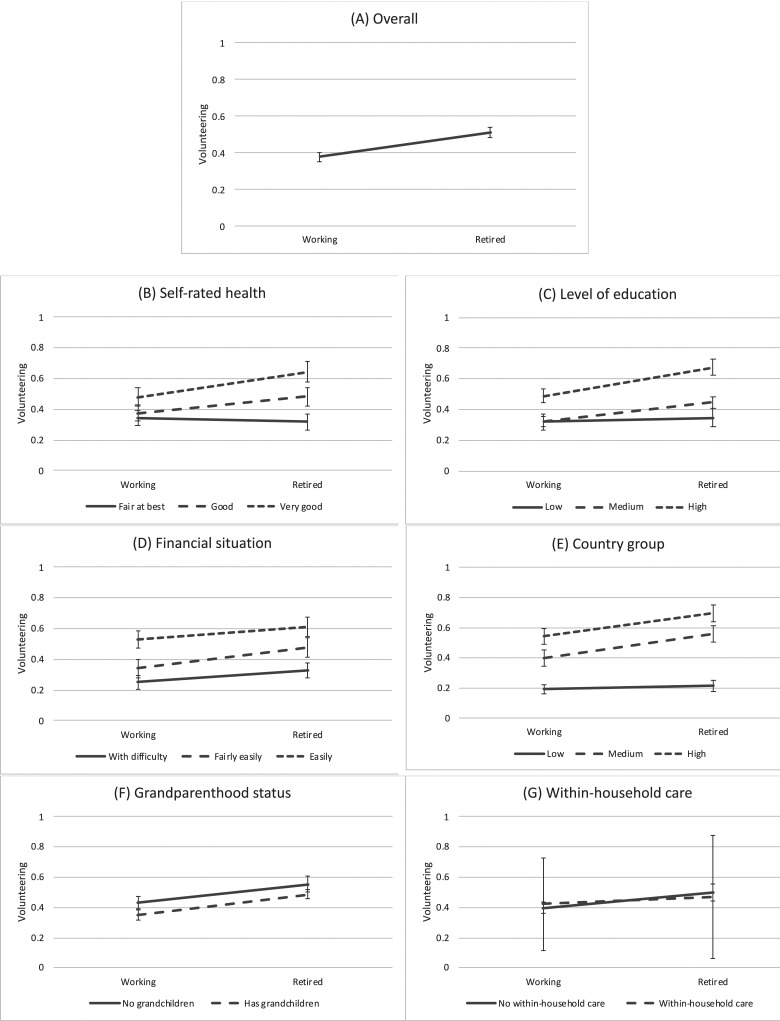
Within-person associations between retirement and frequency of volunteering (predictive margins and 95% CIs).

Table 3 presents the results from the within-person regression models considering the association between transition to retirement and volunteering (the full model is available in Appendix Table 2). Predictive margins calculated from the regression models are illustrated in Figure 1 and statistical details are available in Appendix Table 4. First, our findings showed that the transition to retirement was associated with an increased frequency of volunteering among individuals over time. To inspect possible gender difference we split the data by gender, and examined the effects separately, although the association was similar among both females (Coef = 0.17, *p* < .001, 95% CI = 0.10–0.23) and males (Coef = 0.10, *p* < .001, 95% CI = 0.04–0.16) (results not shown in Tables). We then ran the main model without self-rated health as a control because retirement may affect health (i.e., it might be a mediator); this analysis provided similar results to the main analysis (Coef = 0.13, *p* < .001, 95% CI = 0.09–0.18). As retirement can be a gradual process in which an individual reduces time spend on work before completely transitioning to retirement, we also considered working hours before and after retirement. First, we ran a model excluding respondents who did not do any paid work and introduced an interaction term between retirement and hours of work in the model. Appendix Table 5 shows a positive association between retirement and volunteering (Coef = 0.30, *p* = .003, 95% CI = 0.10–0.49), with a negative interaction effect between retirement and working hours (Coef = −0.01, *p* = .033, 95% CI = −0.1 – -0.0004), meaning that the association between retirement and volunteering is positive among working population, although an increase in the hours of work decrease the strength of the association between retirement and frequency of volunteering. Moreover, we ran multiple models exploring if the association between retirement and volunteering differs according to whether individual retires from part-time or full-time work to part-time or full-time retirement. It was detected that the results are very similar regarding all transitions, although in most cases the differences are statistical insignificant owing to the decreased number of observations in the models (Appendix Table 6).

**Table 3.** Within-Person Associations Between Transition to Retirement and Volunteering, Including Interaction Terms Between Retirement and Resource-Related Factors.

					95% Cl	
		Coef.	SE	*p*	lower	upper
Overall effect	Working	ref.				
	Retired	0.13	0.02	0.000	0.09	0.18
Health	Retired	0.04	0.04	0.223	-0.03	0.11
	Retired x Fair at best	ref.				
	Retired x Good	0.11	0.04	0.005	0.04	0.19
	Retired x Very good	0.12	0.04	0.003	0.04	0.20
Education	Retired	0.04	0.04	0.325	-0.04	0.11
	Retired x Low	ref.				
	Retired x Medium	0.08	0.04	0.029	0.01	0.16
	Retired x High	0.15	0.04	0.000	0.07	0.23
Financial condition	Retired	0.03	0.04	0.422	-0.04	0.10
	Retired x With difficulty	ref.				
	Retired x Fairly easily	0.08	0.04	0.067	-0.01	0.17
	Retired x Easily	0.15	0.04	0.000	0.08	0.23
Country group	Retired	0.02	0.03	0.569	-0.05	0.08
					
	Retired x Low	ref.				
	Retired x Medium	0.13	0.03	0.000	0.06	0.20
	Retired x High	0.15	0.03	0.000	0.09	0.22
Grandparenthood	Retired	0.12	0.03	0.000	0.06	0.18
	Retired x No grandchildren	ref.				
	Retired x Has crandhildren	0.02	0.03	0.525	-0.04	0.08
Within-household care^a^	Retired	0.11	0.04	0.007	0.03	0.20
	Retired x Not providing care	ref.				
	Retired x Providing care	-0.03	0.09	0.707	-0.21	0.14

Notes: All models control for the respondent’s age at interview, partnership status, self-rated health, financial condition, and grandparenthood status; *n* = 12,400 person-observations from 6,200 unique individuals.

^a^*n* = 8,060, person-observations from 4030 unique individuals.

We also restricted the sample to only those participants who volunteered (i.e., the “no volunteering” group was excluded) and detected that the transition to retirement was associated with an increased frequency of volunteering among these individuals as well (Coef = 0.17, *p* < .017, 95% CI = 0.03–0.31).

As the volunteering variable was not normally distributed, we also ran sensitivity analyses with dichotomous variables using logistic regression. Three dichotomous variables were used (Model 1: 0 = no volunteering, 1 = at least some volunteering; Model 2: 0 = less often than monthly, 1 = at least almost monthly; Model 3: 0 = less often than almost every week, 1 = almost daily or every week). Significant associations between the transition to retirement and the increased probability of volunteering were found in all these logistic regression models (Appendix Table 7).

Next, we tested the hypotheses related to other individual resources. First, we introduced an interaction term between retirement and health into the model and detected a significant interaction effect (Table 3). Splitting the data by health status revealed that the transition to retirement was associated with an increased frequency of volunteering among those who rated their health as “good” or “very good” but not among those who considered their health to be “fair at best” (Figure 1(b)). Next, a significant interaction effect was detected between education and retirement (Table 3). According to the data split by education, the transition to retirement was associated with an increased frequency of volunteering among the groups “medium” and “high” but not in the group “low” (Figure 1(c)). Thereafter, we found a significant interaction effect between retirement and financial situation (Table 3). Although the transition to retirement was associated with increased frequency of volunteering among all groups, the better was the financial situation, the higher was the frequency of volunteering and also the effect of transition to retirement on volunteering was slightly stronger among those who considered their household manages financially fairly easily (Figure 1(d)). These findings provided support for Hypothesis 2a, 2b and 2c.

Next, we explored whether the association between retirement and volunteering varies according to countries’ GDP per capita and detected a significant interaction effect (Table 3). Upon splitting the data by country groups, we found that the positive association between retirement and frequency of volunteering was strongest in countries with a high GDP per capita, whereas no significant association was detected among countries with a low GDP per capita (Figure 1(e)), providing evidence for Hypothesis 3.

Finally, we tested whether transition to retirement is associated with increased frequency of volunteering less strongly among individuals with potential family care duties but found no significant interaction effects regarding grandparent status or within-household care provision. Our findings also indicated that those without grandchildren volunteered more than grandparents both before and after retirement (Figure 1(f)) and those without within-household care duties did not volunteer significantly more than those who had such duties (Figure 1(g)). Hence, Hypotheses 4a or 4b were not supported.

## Discussion

Volunteering serves as a potential substitute for employment, providing continuity after retirement. This study investigated whether the transition from employment to retirement is associated with the frequency of volunteering among older Europeans. Prior studies have detected that retired older adults volunteer more compared to those who are still working (Hank & Erlinghagen, 2010a; Mutchler et al., 2003). We, however, focused on changes in the frequency of volunteering before and after retirement within individuals’ lives. In line with a previous study showing a positive association between retirement and volunteering (Eibich et al., 2022), we found that when older adults retire, their contribution to volunteering work increases. The tendency was observed among both females and males, as well as among older adults who started (i.e., changed from the category “no volunteering” to “volunteering”) and intensified (i.e., they had also volunteered before retirement) volunteering after retirement. Moreover, transition to retirement was associated with increased frequency of volunteering among those who continued part-time working after retirement as well as among those who fully retired.

We found several differences in the association between the transition to retirement and volunteering according to factors related to individuals’ resources. Transition to retirement was more strongly associated with an increased frequency of volunteering among those with better self-rated health and among more educated individuals. In addition, a slightly stronger increase in frequency of volunteering after retirement was found among older adults whose household managed financially fairly easily compared to those who experienced financial difficulties. Although prior studies have found that individuals with better health, higher education and improved financial situation tend to volunteer more than individuals with poor health, lower education and worse financial situation, respectively (e.g., Ajrouch et al., 2016; Choi, 2003; Hank, 2011; Principi et al., 2016), the present study showed for the first time that the positive association between retirement and volunteering is stronger among individuals with better resources compared to those with less resources. These findings provided support for the importance of resources influencing the retirement effect and in shaping the behavior of older adults as volunteers.

The transition to retirement exhibited a stronger association with the frequency of volunteering in countries with higher GDP per capita, whereas no significant association was found among countries with the lowest levels of GDP per capita. This result is in line with previous findings showing that older adults volunteer more in countries with better economical well-being and more comprehensive welfare state system (e.g., Hank & Erlinghagen, 2010b; Morawski et al., 2020). However, as an unique contribution to prior studies, our investigation showed that the frequency of volunteering increased more after retirement in countries with higher GDP per capita compared to lower GDP per capita, which highlights the importance of country-level resources moderating retirement effect on volunteering.

We did not find support for the prediction that transition to retirement is associated with increased frequency of volunteering more strongly among older adults without grandchildren or without family caregiving responsibilities. Grandparents volunteered less frequently than non-grandparents both before and after retirement but there was no evidence for significant retirement effect. Moreover, we were unable to find support for significant retirement effects according to within-household care duties.

According to the continuity theory, during their transition to retirement, older adults may seek to maintain continuity in their daily lives by increasing their involvement in volunteering. This engagement can serve as a substitute for the activities and social connections lost upon leaving employment (Chambre, 1984; Eibich et al., 2022; Van den Bogaard et al., 2014). Consistently, we found that transition to retirement was associated with increased frequency of volunteering among older Europeans. Resource theory of volunteering suggests that increased resources are associated with increased rates of volunteering (Wilson & Musick, 1997). In accordance with the resource theory, we detected that, in addition to improved time resources due to retirement, several other individual-level resources (indicated by better health, more advanced education and improved financial condition) were associated with increased frequency of volunteering. Furthermore, our results suggest that in addition to individual-level resources, it is important to also consider country-level resources as the association was stronger in countries with higher GDP per capita than in those with lower GDP per capita.

The present study has several methodological strengths. We used population-based and longitudinal data covering older adults from different parts of Europe. In SHARE, the same individuals were interviewed repeatedly, making it possible to study the frequency of volunteering before and after retirement. To take full advantage of our panel data, we ran within-person regression models, which concentrated on individuals’ varying behaviors over time, and removed all the time-invariant factors. Employing the within-person approach also enhanced us to draw “more causal” interpretations of the association between retirement and volunteering. Finally, the SHARE data allowed us to consider several time-variant factors and investigate whether the transition to retirement is associated with the frequency of volunteering differently according to individual- and country-level resources.

Although the within-person models have several strengths, they also have some limitations. First, the within-person models may include a limited number of participants who showed variations in the outcome measure between study waves; therefore, in some cases, the confidence intervals were quite wide. Second, selective panel attrition is a common condition in longitudinal surveys where the same individuals are interviewed repeatedly. In the present study, selective panel attrition may have been present if the older adults who volunteer frequently may also be most likely to participate in follow-up surveys. This can limit the generalizability of the results, specifically regarding individuals whose volunteering habits remained constant over the waves of data collection. Finally, although the within-person models considered all the time-invariant factors, they did not adjust for unobserved time-variant factors: Although we controlled for several time-variant factors available in the SHARE data in the within-person analyses, it is hard, if not impossible, to take all such factors into account.

## Conclusions

How older adults organize their everyday life after retirement is a salient question in aging societies, especially from the policy perspective. As engagement in unpaid productive activities is a significant predictor of a happy and healthy life in older adults (Burr et al., 2021) and may benefit the overall society (Musick & Wilson, 2007), it is unsurprising that the social engagement of older adults in these activities has been central in many policy debates. The present study shows that individuals may continue being active and productive part of society after they retire. Moreover, our results suggest that improved resources of older adults and economical well-being of a society in general, may promote active ageing.

## Supplemental Material

Supplemental Material - Is Transition to Retirement Associated With Volunteering? Longitudinal Evidence from Europe Supplemental Material for Is Transition to Retirement Associated With Volunteering? Longitudinal Evidence from Europe by Hans Hämäläinen, Antti O. Tanskanen, Bruno Arpino, Aïda Solé-Auró and Mirkka Danielsbacka in Research on Aging

## References

[bibr1-01640275241251786] Ajrouch K. J. Antonucci T. C. Webster N. J. (2016). Volunteerism: Social network dynamics and education. Journals of Gerontology Series B: Psychological Sciences and Social Sciences, 71(2), 309–319. 10.1093/geronb/gbu166 25512570 PMC4817080

[bibr2-01640275241251786] Allison P. D. (2009). Fixed effects regression models. Sage.

[bibr3-01640275241251786] Arpino B. Solé-Auró A. (2019). Education inequalities in health among older European men and women: The role of active aging. Journal of Aging and Health, 31(1), 185–208. 10.1177/0898264317726390 28823184

[bibr4-01640275241251786] Atchley R. C. (1971). Retirement and leisure participation: Continuity or crisis? The Gerontologist, 11(1), 13–17. 10.1093/geront/11.1_part_1.13 5579223

[bibr5-01640275241251786] Atchley R. C. (1989). A continuity theory of normal aging. The Gerontologist, 29(2), 183–190. 10.1093/geront/29.2.183 2519525

[bibr6-01640275241251786] Baik S. Crittenden J. Coleman R. (2023). Social capital and formal volunteering among family and unpaid caregivers of older adults. Research on Aging, 46(2), 127–138. 10.1177/01640275231202260 37714189

[bibr7-01640275241251786] Bergmann M. Kneip T. De Luca G. Scherpenzeel A. (2022). Survey participation in the eighth wave of the survey of health, ageing and retirement in Europe (SHARE). SHARE working paper series 81. 10.17617/2.3390284

[bibr8-01640275241251786] Bethmann A. Bethmann M. Scherpenzeel A. (2019). SHARE sampling guide - wave 8. SHARE-ERIC. SHARE working paper series 33.

[bibr9-01640275241251786] Börsch-Supan A. Brandt M. Hunkler C. Kneip T. Korbmacher J. Malter F. Schaan B. Stuck S. Zuber S. SHARE Central Coordination Team . (2013). Data resource profile: The survey of health, ageing and retirement in Europe (SHARE). International Journal of Epidemiology, 42(4), 992–1001. 10.1093/ije/dyt088 23778574 PMC3780997

[bibr51-01640275241251786] Brady H. E. Schlozman K. L. Verba S. (1999). Prospecting for participants: Rational expectations and the recruitment of political activists. American Political Science Review, 93(1), 153–168. 10.2307/2585767

[bibr10-01640275241251786] Brüderl J. Ludwig V. (2015). Fixed-effects panel regression. In Best H. Wolf C. (Eds.), The Sage handbook of regression analysis and causal inference (pp. 327–358). Sage. 10.4135/9781446288146.n15

[bibr11-01640275241251786] Burr J. A. Mutchler J. E. Han S. H. (2021). Volunteering and health in later life. In Ferraro K. F. Carr D. (Eds.), Handbook of aging and the social sciences (9th ed., pp. 303–319). Academic Press. 10.1016/B978-0-12-815970-5.00019-X

[bibr12-01640275241251786] Butrica B. A. Johnson R. W. Zedlewski S. R. (2009). Volunteer dynamics of older Americans. Journals of Gerontology Series B: Psychological Sciences and Social Sciences, 64(5), 644–655. 10.1093/geronb/gbn042 19213847

[bibr13-01640275241251786] Chambre S. M. (1984). Is volunteering a substitute for role loss in old age? An empirical test of activity theory. The Gerontologist, 24(3), 292–298. 10.1093/geront/24.3.292 6745666

[bibr14-01640275241251786] Cheng G. H. L. Chan A. Østbye T. Malhotra R. (2022). The association of human, social, and cultural capital with prevalent volunteering profiles in late midlife. European Journal of Ageing, 19(1), 95–105. 10.1007/s10433-021-00605-x 35250421 PMC8881546

[bibr15-01640275241251786] Choi L. H. (2003). Factors affecting volunteerism among older adults. Journal of Applied Gerontology, 22(2), 179–196. 10.1177/0733464803022002001

[bibr16-01640275241251786] Choi N. G. Burr J. A. Mutchler J. E. Caro F. G. (2007). Formal and informal volunteer activity and spousal caregiving among older adults. Research on Aging, 29(2), 99–124. 10.1177/0164027506296759

[bibr18-01640275241251786] Curran P. J. Bauer D. J. (2011). The disaggregation of within-person and between-person effects in longitudinal models of change. Annual Review of Psychology, 62, 583–619. 10.1146/annurev.psych.093008.100356 PMC3059070 19575624

[bibr19-01640275241251786] Di Gessa G. Bordone V. Arpino B. (2020). Becoming a grandparent and its effect on well-being: The role of order of transitions, time, and gender. Journals of Gerontology Series B: Psychological Sciences and Social Sciences, 75(10), 2250–2262. 10.1093/geronb/gbz135 31628843 PMC7664312

[bibr20-01640275241251786] Egerton M. Mullan K. (2008). Being a pretty good citizen: An analysis and monetary valuation of formal and informal voluntary work by gender and educational attainment. British Journal of Sociology, 59(1), 145–164. 10.1111/j.1468-4446.2007.00186.x 18321335

[bibr21-01640275241251786] Eibich P. Lorenti A. Mosca I. (2022). Does retirement affect voluntary work provision? Evidence from Europe and the US. Labour Economics, 76, 102185. 10.1016/j.labeco.2022.102185

[bibr22-01640275241251786] Erlinghagen M. (2010). Volunteering after retirement: Evidence from German panel data. European Societies, 12(5), 603–625. 10.1080/14616691003716902

[bibr23-01640275241251786] Erlinghagen M. Hank K. (2006). The participation of older Europeans in volunteer work. Ageing and Society, 26(4), 567–584. 10.1017/S0144686X06004818

[bibr24-01640275241251786] Eurostat (2023). Real GDP per capita. Chain linked volumes (2010), Euro per capita. (Accessed 27.6.2023). https://ec.europa.eu/eurostat/databrowser/view/SDG_08_10__custom_6674071/settings_1/map?lang=en

[bibr25-01640275241251786] Grünwald O. Damman M. Henkens K. (2021). The differential impact of retirement on informal caregiving, volunteering, and grandparenting: Results of a 3-year panel study. Journals of Gerontology Series B: Psychological Sciences and Social Sciences, 76(3), 607–619. 10.1093/geronb/gbaa221 33294930 PMC8611689

[bibr26-01640275241251786] Hämäläinen H. Tanskanen A. O. Danielsbacka M. (2023). Who are ‘multi-helpers’? Profile of older adults engaging in multiple help-giving activities. Journal of Population Ageing. Advanced Online Publication. 10.1007/s12062-023-09415-8

[bibr27-01640275241251786] Hank K. (2011). Societal determinants of productive aging: A multilevel analysis across 11 European countries. European Sociological Review, 27(4), 526–541. 10.1093/esr/jcq023

[bibr28-01640275241251786] Hank K. Erlinghagen M. (2010a). Dynamics of volunteering in older Europeans. The Gerontologist, 50(2), 170–178. 10.1093/geront/gnp122 19666783 PMC2838410

[bibr29-01640275241251786] Hank K. Erlinghagen M. (2010b). Volunteering in “old” Europe: Patterns, potentials, limitations. Journal of Applied Gerontology, 29(1), 3–20. 10.1177/0733464809333884

[bibr30-01640275241251786] Hank K. Stuck S. (2008). Volunteer work, informal help, and care among the 50+ in Europe: Further evidence for ‘linked’productive activities at older ages. Social Science Research, 37(4), 1280–1291. 10.1016/j.ssresearch.2008.03.001 19227703

[bibr31-01640275241251786] Henning G. Arriagada C. Karnick N. (2023). Retirement and volunteering in Germany–historical changes and social inequalities. Research on Aging, 46(1), 15–28. 10.1177/01640275231170798 37066989

[bibr32-01640275241251786] Jokela M. Airaksinen J. Kivimäki M. Hakulinen C. (2018). Is within-individual variation in personality traits associated with changes in health behaviours? Analysis of seven longitudinal cohort studies. European Journal of Personality, 32(6), 642–652. 10.1002/per.2173

[bibr34-01640275241251786] Mood C. (2010). Logistic regression: Why we cannot do what we think we can do, and what we can do about it. European Sociological Review, 26(1), 67–82. 10.1093/esr/jcp006

[bibr35-01640275241251786] Morawski L. Okulicz-Kozaryn A. Strzelecka M. (2020). Elderly volunteering in Europe: The relationship between volunteering and quality of life depends on volunteering rates. Voluntas: International Journal of Voluntary and Nonprofit Organizations, 33(2), 256–268. 10.1007/s11266-020-00267-w

[bibr36-01640275241251786] Morgan S. L. (Ed.), (2013). Handbook of causal analysis for social research. Springer.

[bibr37-01640275241251786] Musick M. A. Wilson J. (2007). Volunteers: A social profile. Indiana University Press.

[bibr38-01640275241251786] Mutchler J. E. Burr J. A. Caro F. G. (2003). From paid worker to volunteer: Leaving the paid workforce and volunteering in later life. Social Forces, 81(4), 1267–1293. 10.1353/sof.2003.0067

[bibr39-01640275241251786] Papa R. Cutuli G. Principi A. Scherer S. (2019). Health and volunteering in Europe: A longitudinal study. Research on Aging, 41(7), 670–696. 10.1177/0164027519834939 30845894

[bibr40-01640275241251786] Principi A. Galenkamp H. Papa R. Socci M. Suanet B. Schmidt A. Schulmann K. Golinowska S. Sowa A. Moreira A. Deeg D. J. H. Deeg D. J. (2016). Do predictors of volunteering in older age differ by health status? European Journal of Ageing, 13(2), 91–102. 10.1007/s10433-016-0377-0 28804374 PMC5550605

[bibr41-01640275241251786] Rotolo T. Wilson J. (2006). Substitute or complement? Spousal influence on volunteering. Journal of Marriage and Family, 68(2), 305–319. 10.1111/j.1741-3737.2006.00254.x, https://www.jstor.org/stable/3838902

[bibr42-01640275241251786] Strauss S. (2021). Multiple engagement: The relationship between informal care-giving and formal volunteering among europe's 50+ population. Ageing and Society, 41(7), 1562–1586. 10.1017/S0144686X19001764

[bibr43-01640275241251786] Tang F. (2016). Retirement patterns and their relationship to volunteering. Nonprofit and Voluntary Sector Quarterly, 45(5), 910–930. 10.1177/0899764015602128

[bibr52-01640275241251786] Tanskanen A. O. Danielsbacka M. (2019). Intergenerational Family Relations: An Evolutionary Social Science Approach. New York & London: Routledge.

[bibr44-01640275241251786] Tanskanen A. O. Danielsbacka M. Hämäläinen H. Solé-Auró A. (2021). Does transition to retirement promote grandchild care? Evidence from Europe. Frontiers in Psychology, 12, 738117. 10.3389/fpsyg.2021.738117 34616345 PMC8489495

[bibr45-01640275241251786] Tanskanen A. O. Hämäläinen H. Arpino B. Danielsbacka M. (2022). Prosocial activity in later life: Are informal help and care associated with volunteering and charity? Ageing and Society, 1, 1–36. 10.1017/S0144686X22001015

[bibr46-01640275241251786] Van den Bogaard L. Henkens K. Kalmijn M. (2014). So now what? Effects of retirement on civic engagement. Ageing and Society, 34(7), 1170–1192. 10.1017/S0144686X13000019

[bibr47-01640275241251786] Walker A. Maltby T. (2012). Active ageing: A strategic policy solution to demographic ageing in the European union. International Journal of Social Welfare, 21(S1), 117–130. 10.1111/j.1468-2397.2012.00871.x

[bibr48-01640275241251786] Williams R. (2012). Using the margins command to estimate and interpret adjusted predictions and marginal effects. STATA Journal: Promoting communications on statistics and Stata, 12(2), 308–331. 10.1177/1536867X1201200209

[bibr49-01640275241251786] Wilson J. Musick M. (1997). Who cares? Toward an integrated theory of volunteer work. American Sociological Review, 62(5), 694–713. 10.2307/2657355

[bibr50-01640275241251786] Wilson J. Musick M. (1998). The contribution of social resources to volunteering. Social Science Quarterly, 799–814. https://www.jstor.org/stable/42863848

