# Supplementary material for: Is Transition to Retirement Associated With Volunteering? Longitudinal Evidence from Europe
Source: Res Aging. 2024 May 10;46(9-10):509–20. doi: 10.1177/01640275241251786 (PMC11423552; doi:10.1177/01640275241251786)
Supplement: Supplemental Material - Is Transition to Retirement Associated With Volunteering? Longitudinal Evidence from Europe [file sj-pdf-1-roa-10.1177_01640275241251786.pdf]

Appendix Table 1. Transitions in the frequency of volunteering.

	Time 2					Total n
	0	1	2	3	4	
Time 1						
0 No volunteering	4,506	174	206	213	64	5,163
1 Less than monthly	133	46	32	38	6	255
2 Almost every month	131	18	88	88	9	334
3 Almost every week	112	15	41	125	49	342
4 Almost daily	34	3	7	24	38	106
Total n	4,916	256	374	488	166	6,200

Appendix Table 2. Within-person associations between time-variant factors and volunteering.

				95% Cl	
	Coef.	SE	p	lower	upper
**Retirement status**					
Working	ref.				
Retired	0.13	0.02	0.000	0.09	0.18
**Age at interview**	-0.002	0.01	0.781	-0.02	0.01
**Self-rated health**					
Fair at best	ref.				
Good	-0.06	0.03	0.027	-0.12	-0.01
Very good	-0.04	0.04	0.269	-0.11	0.03
**Partnership status**					
Living without partner	ref.				
Living with partner	0.06	0.09	0.555	-0.13	0.24
**Financial condition**					
With difficulty	ref.				
Fairly easily	0.02	0.03	0.604	-0.04	0.07
Easily	0.01	0.03	0.881	-0.06	0.07
**Grandparenthood status**					
No grandchildren	ref.				
Has grandchildren	0.04	0.05	0.429	-0.06	0.14

Notes: n = 12,400 person-observations from 6,200 unique individuals.

Appendix Table 3. Within-person associations between time-variant factors including within-household care and volunteering.

	Coef.	SE	p	95% Cl lower	upper
**Retirement status**					
Working	ref.				
Retired	0.11	0.04	0.007	0.03	0.19
**Age at interview**	0.01	0.02	0.609	-0.02	0.04
**Self-rated health**					
Fair at best	ref.				
Good	-0.01	0.04	0.752	-0.08	0.06
Very good	0.02	0.05	0.670	-0.07	0.11
**Partnership status**					
Living without partner	ref.				
Living with partner	0.14	0.14	0.320	-0.14	0.42
**Financial condition**					
With difficulty	ref.				
Fairly easily	-0.04	0.04	0.272	-0.11	0.03
Easily	-0.03	0.04	0.512	-0.11	0.05
**Grandparenthood status**					
No grandchildren	ref.				
Has grandchildren	0.03	0.05	0.636	-0.08	0.13
**Provided care within household**					
No	ref.				
Yes	0.08	0.06	0.192	-0.04	0.21

Notes: n = 8,060 person-observations from 4,030 unique individuals.

Appendix Table 4. Predictive margins of the associations between transition to retirement and volunteering.

			95% Cl	
		Margin	lower	upper
**Overall effect**				
	Working	0.38	0.35	0.40
	Retired	0.51	0.48	0.54
**Self-rated health**				
Fair at best	Working	0.34	0.29	0.39
	Retired	0.32	0.27	0.37
Good	Working	0.37	0.32	0.43
	Retired	0.48	0.42	0.54
Very good	Working	0.48	0.42	0.54
	Retired	0.64	0.58	0.71
**Education**				
Low	Working	0.32	0.27	0.37
	Retired	0.35	0.29	0.40
Medium	Working	0.32	0.28	0.35
	Retired	0.44	0.41	0.48
High	Working	0.49	0.44	0.53
	Retired	0.67	0.62	0.72
**Financial condition**				
With difficulty	Working	0.25	0.21	0.30
	Retired	0.33	0.28	0.38
Fairly easily	Working	0.34	0.28	0.40
	Retired	0.48	0.41	0.54
Easily	Working	0.53	0.47	0.59
	Retired	0.61	0.55	0.68
**Country groups**				
Low	Working	0.19	0.16	0.22
	Retired	0.21	0.18	0.25
Medium	Working	0.40	0.35	0.45
	Retired	0.56	0.50	0.61
High	Working	0.54	0.49	0.60
	Retired	0.70	0.64	0.75

**Grandparenthood status**				
No grandchildren	Working	0.43	0.39	0.47
	Retired	0.55	0.50	0.61
Has grandchildren	Working	0.35	0.32	0.38
	Retired	0.49	0.45	0.52
**Provided care within household** [1]				
No	Working	0.40	0.36	0.43
	Retired	0.50	0.44	0.55
Yes	Working	0.42	0.11	0.73
	Retired	0.47	0.06	0.87
**Partnership status**				
Living without partner	Working	0.38	0.33	0.44
	Retired	0.51	0.45	0.57
Living with partner	Working	0.37	0.35	0.40
	Retired	0.51	0.48	0.54

Notes: n = 12,400 person-observations from 6,200 unique individuals.

[1] n = 8,060, person-observations from 4,030 unique individuals.

person-observations from 5,247 unique individuals.

Appendix Table 5. Within-person association between transition to retirement and volunteering among working individuals, including interaction term between retirement and weekly working hours.

					95% Cl	
		Coef.	SE	p	lower	upper
**Overall effect**	Working	ref.				
	Retired	0.14	0.07	0.034	0.01	0.27
**Working hours per week**	Retired	0.30	0.10	0.003	0.10	0.49
	Retired x Working hours	-0.01	0.00	0.033	-0.01	-0.0004

Notes: Respondents who work at least 1 hour per week; n = 2,558 person-observations from 1,279 unique individuals.
Models control for the respondent's age at interview, partnership status, self-rated health, financial condition, and grandparenthood status.

Appendix Table 6. Within-person associations between transition to retirement and volunteering with information on working hours.

					95% Cl	
		coef.	SE	p	lower	upper
Transition to full retirement						
Model 1a	Work max. 20h	ref.				
	Retired	0.06	0.06	0.359	-0.07	0.18
Model 1b	Work over 20h	ref.				
	Retired	0.17	0.04	0.000	0.09	0.24
Transition to part-time retirement (work max. 20h / week)						
Model 2a	Work max. 20h	ref.				
	Partly retired (max. 20h work)	0.21	0.09	0.026	0.03	0.40
Model 2b	Work over 20h	ref.				
	Partly retired (max. 20h work)	0.12	0.12	0.308	-0.11	0.35
Transition to part-time retirement (work over 20h / week)						
Model 3a	Work max. 20h	ref.				
	Partly retired (over 20h work)	0.12	0.17	0.466	-0.21	0.45
Model 3b	Work over 20h	ref.				
	Partly retired (over 20h work)	0.11	0.08	0.172	-0.05	0.27

Note.

Model 1a: 1,658 observations from 829 individuals. Model 1b: 7,650 observations from 3,825 individuals.

Model 2a: 528 observations from 264 individuals. Model 2b: 510 observations from 255 individuals.

Model 3a: 240 observations from 120 individuals. Model 3b: 1,556 observations from 778 individuals.
All models control for the respondent’s age at interview, partnership status, self-rated health, financial condition, and grandparenthood status.

Appendix Table 7. Within-person associations between the transition to retirement and volunteering with dichotomous volunteering variables.

	Odds ratio	SE	p	95% Cl lower	upper
Model 1					
Working	ref				
Retired	1.70	0.19	0.000	1.38	2.11
Model 2					
Working	ref				
Retired	1.81	0.22	0.000	1.43	2.30
Model 3					
Working	ref				
Retired	2.05	0.29	0.000	1.55	2.71

Notes: Model 1: No volunteering vs. At least some volunteering; n = 2,134 person-observations from 1,067 unique individuals; Model 2: Less often than monthly vs. monthly volunteering; n = 1,744 person-observations from 872 unique individuals; Model 3: Less often than weekly vs. weekly volunteering; n = 1,260 person-observations from 630 unique individuals; All models control for the following time-variant factors: respondent's age at interview, partnership status, self-rated health, financial condition, and grandparenthood status.
